# Characterization of a New Lightened Gypsum-Based Material Reinforced with Fibers

**DOI:** 10.3390/ma14051203

**Published:** 2021-03-04

**Authors:** Manuel Álvarez, Daniel Ferrández, Carlos Morón, Evangelina Atanes-Sánchez

**Affiliations:** 1 Department of Building Technologies, Higher Technical School of Building, Universidad Politécnica de Madrid, 28040 Madrid, Spain; manuel.alvarezd@upm.es (M.Á.); daniel.fvega@upm.es (D.F.); 2 Department of Organization Engineering, Business Administration and Statistics, Higher Technical School of Industrial Engineering, Universidad Politécnica de Madrid, 28660 Madrid, Spain; 3 Department of Mechanical & Chemical Engineering and Industrial Design, Higher Technical School of Industrial Engineering and Design, Universidad Politécnica de Madrid, 28012 Madrid, Spain; evangelina.atanes@upm.es

**Keywords:** lightened plaster, reinforcing fibers, polymers, precast materials

## Abstract

This paper shows the characterization of a new lightened gypsum-based material for use in buildings. A plaster material has been designed with a polymeric compound based on polyvinyl acetate, bicarbonate and a boric acid solution, which reduce the density and thermal conductivity by up to 20% and 30%, respectively. In addition, tests have been carried out with the lightened plaster material reinforced with glass (GF), basalt (BF), polypropylene (PPF) and wood (WF) fibers. A significant improvement in mechanical properties was achieved. All samples obtained resistance values greater than 2 MPa in flexion and 3 MPa in compression. Physico-chemical analysis were also carried out. The study is completed with a statistical analysis, where confidence intervals have been obtained for the mean at 95% confidence for each of the physical properties studied.

## 1. Introduction

Gypsum has been one of the most widely used construction materials since ancient times. Its main raw material is gypsum rock, which is found in nature in the form of sedimentary rock of chemical precipitation constituted by calcium sulfate dihydrate $\left({\mathrm{CaSO}}_{4}\cdot 2H_{2}O\right)$ [1]. The main reasons for its widespread application in construction are its easy removal, its versatility in use and its rapid implementation. It is also a low-cost material, with a clean attractive finish [2].

One of the most relevant properties of construction plaster includes the exothermic reaction that plaster powder experiences when it comes into contact with water. This causes a rapid setting of the material [3]. Adhesion capacity to ceramic materials and a high capacity of hygrothermal regulation are also properties that make it an ideal interior coating [4]. High surface hardness that evolves inversely with the water content and its good acoustic and fireproof insulation also contribute to its great performance in buildings. It also presents thermal conductivity values between 0.2 and 0.6 W/mK [5]. However, the high industrialization of the building sector, and the rise in increasingly demanding technical requirements, have led multiple researchers to carry out studies that allow the functions of plaster-based materials to be improved or adapted to these needs, in particular, with regard to the preparation of plaster precasts for construction [6,7].

In this respect, several authors have experimented with different compositions to lighten the weight of gypsum-based materials. Gencel et al. [8] have developed a new gypsum-based compound with the addition of diatomite and polypropylene fibers. This addition manages to reduce the density of the hardened material by improving its thermal conductivity. Another widely known method to lighten plaster precasts involves the addition of expanded polystyrene or cork granules in the matrix [9,10]. Along these lines, Bicer and Kar have experimented with the combined incorporation of expanded polystyrene and tragacanth. This creates a network of internal pores in the mass of the material to reduce the weight of the precast blocks [11]. Materials such as perlite and vermiculite have also been used as additions to lighten the weight of plaster-based materials. Evans et al. [12] developed a new method for the preparation of semi-dry plaster mortars, which incorporated expanded perlite with glass fibers. The fact that these lightened plaster materials are easily applicable on site and have good thermal resistance leads to their incorporation as rehabilitation materials, even improving the results obtained with porous materials made with Portland cement [13]. Finally, we find authors who have focused their research on reducing the weight of plaster materials based on circular economy criteria and the incorporation of waste, such as the addition of polyamide powder waste or plastic cable waste [14,15].

However, generally, the techniques used to lighten the weight of building conglomerate materials are associated with a reduction in the final mechanical properties [16]. For this reason, several studies focus on the incorporation of fibers to improve physical performance. These fibers can be natural or synthetic. Natural fibers are more environmentally friendly as they are produced without human intervention and a smaller number of chemical treatments [17].

A wide variety of natural fibers are used as reinforcing material, including wood, straw, coconut, banana, animal hair and palm fibers [18,19,20]. The amount of fibers used is also a discriminant that must be set experimentally, as recent studies, such as that of Dai and Fan [21], have shown that with additions between 20% and 30% of wood sawdust, the optimum quantity for reinforcement was obtained. This produces a decrease in the final mechanical properties of the material. This decrease in mechanical properties after an excessive incorporation of fibers in the matrix has been confirmed in several studies [22]. On the other hand, tests have been carried out by incorporating previously treated hemp fiber as the reinforcing material to prevent brittle breakage of the prefabricated plaster plates, obtaining strengths similar to those achieved with fibers of synthetic origin [23]. Thus, it can be seen that, in general, natural fibers have greater water absorption during kneading, greater hygrothermal regulation and a greater risk of degradation by decomposition [24,25].

Studies focused on synthetic fibers are also found. Polypropylene, glass, aramid or carbon fibers have been studied, of which the last two are rarely used for the reinforcement of plaster materials due to their high cost [26]. Suarez et al. [27] have carried out studies with the addition of macro- and micropolymeric fibers of different lengths, showing that there is an optimal length for each type of fiber by which the workability of the material is reduced in the fresh state. The addition of macropolymeric fibers gives rise to materials with better mechanical resistance. This decrease in the workability of the material and an increase in the strength of the hardened material have been corroborated by Zhu et al., through the addition of polyvinyl alcohol and polypropylene fibers in gypsum composites [28]. It should be noted that there are studies that try to combine different types of reinforcing fibers to check whether they act in a synergistic mutually beneficial manner [29]. This is the case of recent investigations with additions of polypropylene fiber and isostatic graphite filler (IGF) at 0.6% by volume and 25% by weight, respectively, which have shown better mechanical and thermal results and a greater decrease in porosity when acting together [30].

In recent years, several researchers have focused their work on the study of plaster precasts for buildings [31]. In this sense, authors such as del Rio et al. have been working for years on incorporating construction and demolition waste using sustainability criteria that lead to the recycling of these precasts and other construction materials, such as recycled aggregates [32,33]. However, recent lines of research tend to unite energy efficiency and recycling, as with the investigations carried out by Sair et al., who have recently developed a new eco-friendly composite material based on gypsum reinforced with a mixture of cork fiber and cardboard waste for building thermal insulation [34]. The results show the technical feasibility of applying this material with improved compression resistance and acoustic insulation properties.

The objective of this work is to study the behavior of a new lightened plaster material by adding a polymeric compound based on polyvinyl acetate (PVAc) for its application in buildings and, especially, for the preparation of precast construction. It is a whole new material patented by the researchers with different properties from other lightened gypsum-based materials. An experimental study in which a chemical and physical characterization of the new designed plaster material has been carried out, studying the influence of the addition of four different types of plaster on the mechanical behavior of the material. The reinforcing fibers are glass, basalt, polypropylene and wood.

## 2. Materials and Methods

### 2.1. Experimental Procedure

To carry out this study, an experimental campaign divided into three complementary parts has been carried out. In this way, physico-chemical characterization tests and physical–mechanical characterization tests have been carried out, to finally develop a discussion of the results supported by statistical analysis.

Regarding the physico-chemical characterization, the following tests were carried out: chemical composition by X-ray fluorescence spectrometry from Philips, Madrid, Spain (XRF), simultaneous thermal analysis (STA) with differential scanning calorimetry (DSC) and thermogravimetric analysis from TA Instrument, Madrid, Spain (TGA), X-ray diffraction from Siemens, Madrid, Spain (XRD) and scanning through a light microscope from Philips, Madrid, Spain. The chemical composition of E-35 plaster was analyzed using a Philips-Magic 1 kW XRF spectrometer. A TA Instruments SDT Q600 analysis unit was used for the DSC/TGA test. This analysis was carried out in a range of temperatures between room temperature and 900 °C with increments of 5 °C/minute, under a 100 cm^3^/min flow of air and analyzing a sample mass of approximately 40–50 mg. Regarding the XRD spectra, these were obtained using Siemens Krystalloflex D5000 equipment, with a Cu-Kα graphite monochromator [35]. The diffractograms were obtained in a range of 5° ≤ 2θ ≤ 100° every 0.04°, 4 s per step. The microscopy tests were carried out with an XL 30 ESEM Philips environmental light microscope on the fractured face of the specimens obtained after the flexural test.

For the determination of the physico-mechanical properties of the new lightened plaster material, the tests shown in Table 1 were carried out. It should be noted that all the test pieces included in Table 1 were cured in a laboratory atmosphere at 23 °C and 60% relative humidity. Furthermore, all the samples except those corresponding to the adherence test had been previously dried in an oven at a temperature of 40 °C for 24 h.

Finally, a statistical analysis was carried out to compare the mean values of the different physical and mechanical properties in the different dosages used. For this, non-parametric mean comparison tests were used, specifically the Kruskal–Wallis test for independent samples. Intervals for the mean at a 95% confidence level have been built for each of the properties in each of the samples.

### 2.2. Materials and Dosages Used

The materials used were as follows: plaster, white glue from Supertite (purchased in Madrid, Spain) and hereinafter referred to as polyvinyl acetate or PVAc), sodium bicarbonate, boric acid solution from the Reig-Jofré laboratory (Madrid, Spain), water, synthetic fibers and natural fibers.

#### 2.2.1. Conglomerates

The binder material used in this study was E-35 construction plaster. The basis of the firing of this type of material for its use in construction is carried out through the following basic reaction scheme, which starts as the initial raw material of gypsum rock or natural gypsum stone with the chemical formula CaSO_4_ · 2H_2_O, called calcium sulfate dihydrate (DH):

(1) $${\mathrm{CaSO}}_{4}\cdot 2H_{2}O\to {\mathrm{CaSO}}_{4}\cdot \frac{1}{2}H_{2}O\;\left(\alpha ,\beta \right)+\frac{3}{2}H_{2}O$$

(2) $${\mathrm{CaSO}}_{4}\cdot \frac{1}{2}H_{2}O\to {\mathrm{CaSO}}_{4}+\frac{1}{2}H_{2}O$$

At temperatures around 105–107 °C, DH molecules dehydrate following an endothermic process to obtain mostly calcium sulfate hemihydrate (HH) with the chemical formula CaSO_4_·½H_2_O, in its α (more compact and more resistant) or β (more soluble and less stable) form, the majority component of construction plaster. The rehydration of the HH with the mixing water during its implementation gives rise again to DH, called by some authors calcium sulfate rehydrate (RH) to distinguish it from the natural dihydrate. For its part, HH also undergoes endothermic dehydration to obtain soluble anhydrite or anhydrite III with the chemical formula CaSO_4_ at temperatures close to 200 °C [35]. The exact temperature at which these dehydration reactions take place depends on the experimental conditions, improving when low heating rates are used [39]. The exothermic phase transformation of soluble anhydrite to insoluble anhydrite (anhydrite II), that does not entail associated mass loss, occurs at a temperature around 200 °C, in the case of HH-α and between 300–400 °C for HH-β [40].

For the specific case in this work, an Iberyola E-35 type plaster supplied by PLACO Saint-Gobain (Madrid, Spain) was used, the characteristics of which are shown in Table 2. The chemical composition is 99.7% calcium sulfate, and trace elements of Sr (0.157%), Si (0.068%), Fe (0.035%), Al (0.022%) and P (0.01%)

**Figure 1:**
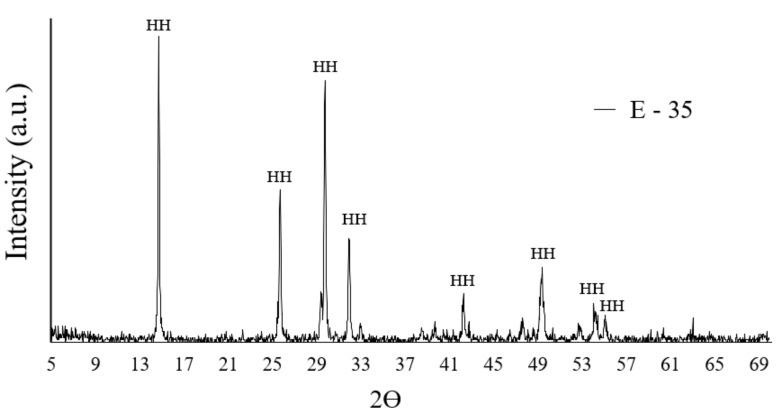
X-ray diffractogram of the plaster E-35 used. HH—calcium sulfate hemihydrate.

Figure 1 shows the XRD analysis of the E-35 plaster powder sample used in this study, where the diffraction peaks corresponding to HH can be seen, according to the bibliography [35,41].

**Figure 2:**
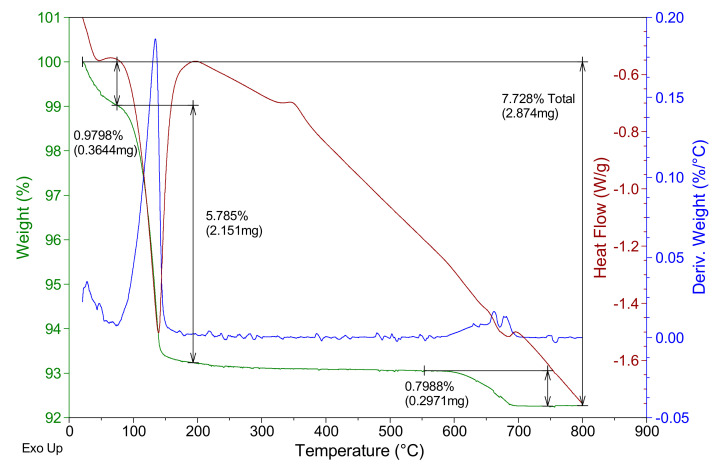
E-35 gypsum thermogravimetric analysis.

In Figure 2, the thermogravimetric analysis of the E-35 plaster used is shown, where the mass of the sample is presented as a percentage and the first derivative of the mass with respect to temperature and the associated heat flux (with the peak-up criterion for an exothermic process) are in green, blue and red, respectively.

In the analysis of Figure 2, an initial loss of mass close to 0.98% can be seen. This occurs at temperatures below 80 °C due to the loss of physically bound water present in the raw material. At 75–180 °C, a second mass loss can be seen which corresponds to the endothermic dehydration of HH to obtain soluble anhydrite, according to Equation (2), with a maximum close to 130 °C, representing a mass loss of 5.7–5.9%. At a temperature of 344 °C, the exothermic transformation of the soluble anhydrite phase to the insoluble anhydrite phase can be observed, without associated mass loss, as corresponds to the β-hemihydrate gypsum. In the range 550–700 °C, there is a mass loss of approximately 0.8%, which would correspond to the endothermic decomposition of some non-identified additives present in the raw material gypsum used in this work.

#### 2.2.2. Polymeric Compound

A mixture of compounds has been added to the main composition of plaster and water, which are responsible for the final lightening of the developed material. It should be noted that hereinafter the mixture of these compounds is called a polymeric compound, consisting of the following: polyvinyl acetate, boric acid solution and sodium bicarbonate.

Polyvinyl acetate dispersions belong to the family of vinyl polymers of a thermoplastic nature. This is a biodegradable, biocompatible, non-toxic polymer that is widely used in the production of adhesives [41]. Thus, polyvinyl acetate has a composition based on a polyvinyl chain with side groups of the acetate type (PVA) (-COO-CH_3_). It is a material that improves the adherence and elasticity of plaster-based materials, hardening by physical means and producing connections between the dihydrate nuclei [42,43]. Its characteristics include its low cost, low toxicity, good creep, thermal stability at room temperature and limited resistance in humid environments, and its use in exteriors is not recommended.

On the other hand, the boric acid solution used is characterized by being isotonic and sterile. Said aqueous solution contains in its composition edetate disodium, poloxamine, sodium borate and sodium chloride. The presence of borate ions in solutions of polyvinyl acetate (PVAc) and/or polyvinyl alcohol (PVA) gives rise to the formation of a three-dimensional semisolid gel, in an exothermic process that entails a large increase in viscosity, caused by crosslinking of polymeric chains [43]. Finally, sodium bicarbonate (NaHCO_3_) is a low-cost, off-white crystalline compound supplied as a powder suitable for dissolution in water. The sodium bicarbonate generates carbon dioxide in an acid medium, in this case provided by the boric acid.

#### 2.2.3. Fibers

For the elaboration of this study, four different types of fibers have been used, which can be classified according to whether their origin is synthetic or natural. The synthetic fibers used are: alkali-resistant glass fiber whose main components are: SiO_2_ (62%), ZrO_2_ (17.3%) and Na_2_O (14.30%), basalt fiber chemically composed of: SiO_2_ (47%), Al_2_O_3_ (13%), CaO (12%), MgO (10%) and polypropylene fiber [44,45,46], supplied by the company SIKA, Madrid, Spain. The natural fiber used is wood fiber from Scots pine consisting of sawdust fibers supplied by Salesianos Carabanchel High School, Madrid, Spain. The main characteristics of the fibers used are shown in Table 3.

#### 2.2.4. Water

The water used in this study is potable water from the Canal de Isabel II in Madrid, in the Community of Madrid. This water does not contain any agent that modifies or alters the properties of the final compound tested [47]. This water has the following properties: middle hardness (25 mg CaCO_3_/L); pH between 7 and 8.5 [48]; chloride content between 1 and 1.5 mg/L. It also contains other compounds, such as: nitrate (0.6 mg/L), nitrite (<0.05 mg/L), sulfate (5.3 mg/L), calcium (17.8 mg/L), iron (0.01 mg/L) and copper (<0.005 mg/L).

#### 2.2.5. Dosages

To obtain the optimal water/binder ratio, the shaking table method included in the UNE-EN-13279-2: 2014 standard [37] was used. Thus, to achieve a diameter of 165 ± 5 mm corresponding to a plastic and workable consistency, a water/plaster mass ratio of 0.66/1 was used.

The notation used to describe the different mixes used in the study use the following coding: D-F, where D indicates the type of polymeric compound addition used, which can be 1 or 2, and F indicates the type of reinforcement used, which can be: fiber-free (NF), fiberglass (GF), basalt fiber (BF), polypropylene fiber (PPF) or wood fiber (WF). In addition, a reference test piece (REF) was made without polymeric compound.

Table 4 shows the different dosages used in this study. It should be noted that all the specimens made for each test come from the same batch in order to obtain uniform and comparable results. Finally, all the samples from the different specimens were taken following the same technique and methods described in the UNE-EN-13279-2: 2014 standard [37].

The amount of fibers added in Table 4 corresponds to an incorporation of 1% by volume of the mixture. Furthermore, prior to kneading, a manual separation of the fibers was carried out to guarantee their homogeneous distribution in the plaster material produced.

The elaboration process for this new material is the following: first of all, mix polyvinyl acetate and water to get a homogeneous liquid; (2) add the boric acid solution and sodium bicarbonate, mixing it carefully; and (3) add the gypsum powder and elaborate the plaster following the UNE-EN-13279-2: 2014 standard [37].

It should be noted that during the preparation of the D1 and D2 series specimens, there was a very large increase in the volume inside the mold, as well as the evolution of heat, which reveals the reaction of the borate ions with the polyvinyl compound as well as the liberation of CO_2_ due to the reaction between sodium bicarbonate and the boric acid.

## 3. Results and Discussion

### 3.1. Physical–Chemical Characterization

**Figure 3:**
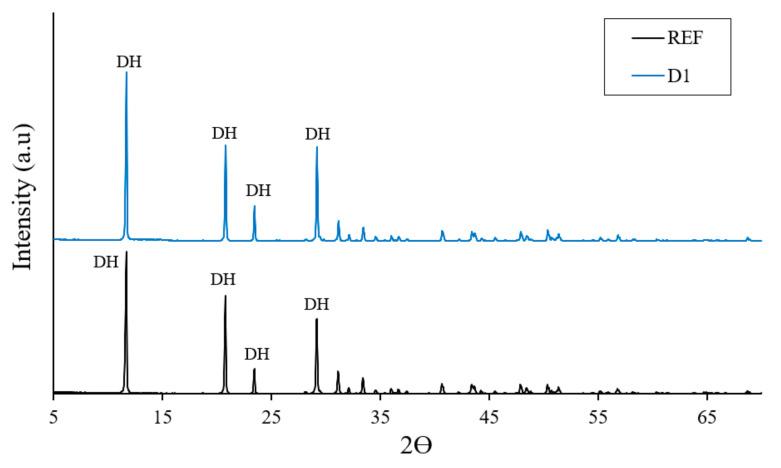
X-ray diffractogram of the reference specimen compared to the lightened specimen with a higher content of polymeric compound and without fibers, where DH refers to calcium sulfate dihydrate.

The X-ray diffraction analysis of the reference and lightened plaster specimens with a higher content of polymeric compound is shown in Figure 3.

The diffractograms in Figure 3 show that both samples present a similar profile in terms of the location of the peaks corresponding to the gypsum dihydrate [49], although it is true that the intensities obtained for the peaks of the lightened plaster sample are greater than in the reference sample.

**Figure 4:**
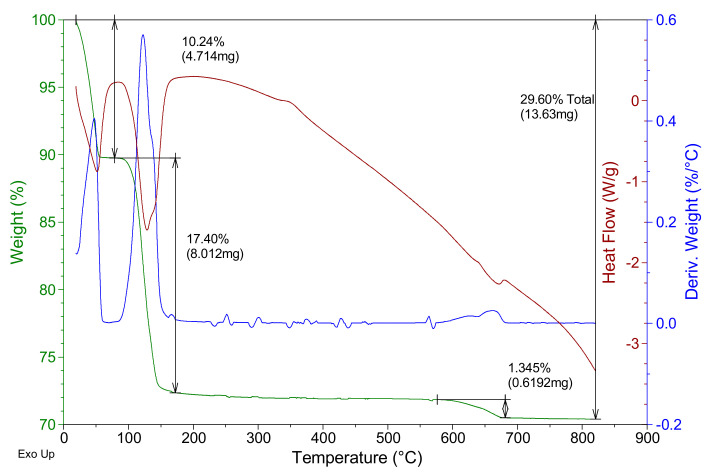
Thermogravimetric analysis of the reference plaster sample.

The thermogravimetric analysis of the reference plaster specimen represented in Figure 4 shows three mass losses, all of them corresponding to endothermic reactions. The first corresponds to the loss of physically bound water, at a temperature below 100 °C, with a maximum at approximately 47 °C. In the temperature interval of 75–175 °C, the second mass loss occurs, with a maximum at 122 °C. This second loss presents a shoulder, with a maximum at approximately 137 °C. The second mass loss is due to the dehydration of the calcium sulfate dihydrate formed in the reaction of the raw material calcium sulfate hemihydrate with the mixing water used in the specimen preparation (according to Equation (1)). The shoulder is due to the dehydration of the formed hemihydrate in the second mass loss (according to Equation (2)). The figure shows, as in the raw material gypsum of Figure 2, the transition phase from soluble to insoluble anhydrite, with a maximum at a temperature of 348 °C, and the fourth mass loss in the range 550–700 °C corresponding to thermal decomposition of additives present in the E-35 gypsum raw material, as seen in Figure 2.

**Figure 5:**
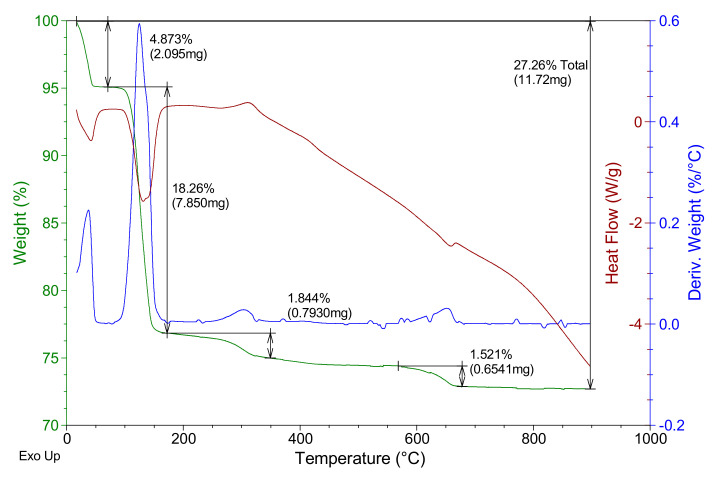
Thermogravimetric analysis of D1 type sample.

Regarding the thermogravimetric analysis of the lightened plaster sample collected in Figure 5, and compared with the reference test piece, the same thermal events are observed with similar numerical values in terms of the value of the mass losses of each process, except for the loss of free water mass. In the lightened specimen, this value is less than 5%, while in the reference specimen, it is around 10%. It should also be considered that the phase transition to soluble anhydrite occurs at a slightly higher temperature in the lightened sample and is seen more diffusely. Additionally, in the lightened plaster sample, a fourth loss of approximately 1.8% by weight can be seen, occurring at temperatures between 200 and 400 °C, with a maximum around 305 °C, and which corresponds to an exothermic reaction. This mass loss can be associated with the thermal decomposition in air atmosphere of the polyvinyl compound used in the formulation [50,51].

### 3.2. Physical–Mechanical Characterization

Firstly, Table 5 shows the results of the test for the determination of the setting start time using the Vicat needle method, as it is the most commonly used procedure to determine this property in plaster materials [52].

As can be deduced from the analysis of Table 5, the batches with the incorporation of the polymeric compound presented setting initiation times that were longer than the reference sample. Thus, the designed material permits an increase in the application time and its workability.

As documented in the literature [43,51], the reaction of borate ions with polyvinyl polymers gives rise to the formation of a three-dimensional network of cross-linked polymeric chains, between which part of the solvent would be trapped, in this case, the mixing water. In this way, the start of the water–hemihydrate hydration reaction to form calcium sulfate dihydrate would be delayed, a reaction responsible for the setting of the gypsum and its mechanical properties, due to the greater difficulty of water in accessing the hemihydrate particles. This would account for the longer setting time observed experimentally for the specimens that incorporate polymer, this delay being greater in the D1 formulation which incorporates a greater amount of polymer.

On the other hand, Table 6 shows the results obtained in the adhesion tests, surface hardness and the actual density of the samples.

Table 6 shows how the batches made with the type D2 dosage had lower adhesion values and greater surface hardness than the type D1 batches, all of them lower than the values presented by the reference specimen. It also shows how the addition of fibers has not led to a significant improvement in surface hardness, and even worsened the adherence resistance of the tested material. However, it should be noted that this decrease in the adhesion values would not be limiting since the new plaster material developed in this research is specially designed for use in the preparation of precasts. On the other hand, the significant decrease in density produced by the incorporation of the polymeric compound during kneading can be seen with respect to the density of the reference specimen, in such a way that, in type D1 and D2 mixes, density decreases of over 22% and 12%, respectively, have been achieved in all the cases analyzed. This decrease in density is maintained in all the specimens that incorporate fibers.

The lower density values for D1 and D2 samples compared with the reference are probably due to their higher porosity compared with the reference. This porosity is originated by the CO_2_ bubble formation during the mass preparation due to the reaction of sodium bicarbonate with acid medium provided by the boric acid solution. These gas bubbles leave the mass, causing voids in the final material and hence a porous structure. The higher proportion of both sodium bicarbonate and boric acid in the D1 series would account for the large quantity of CO_2_ that evolved, and therefore a higher porosity and consequently less density compared with the D2 series.

Additionally, the three-dimensional network formed by the vinyl polymer–borate ion system during the preparation of the specimens could contribute to the higher porosity of D1 and D2 samples compared with the reference. This large volume network would cause the dihydrate particles to be physically further apart from each other, not only during the preparation of the specimens, but also once the structure has been consolidated after drying. This expanded arrangement of the dihydrate particles would cause a higher porosity, and therefore a lower density, of the polymer specimens with respect to the reference sample and, in turn, a higher proportion of polymer and boric acid solution in sample D1, and therefore a greater extension of the three-dimensional network, giving rise to a density lower than D2.

To test how this material reacts to water, a water absorption test by capillarity has been carried out for 10 min in previously dried 4 × 4 × 16 cm^3^ prismatic specimens. It was observed how traditional plasters (REF) absorb up to 17% of their weight in water. On the other hand, type D1 dosages absorb 26% and type D2 22% of their weight in water. In this way, it can be said that the water absorption is greater in the case of the lightened specimens, mainly due to their greater porosity.

**Figure 6:**
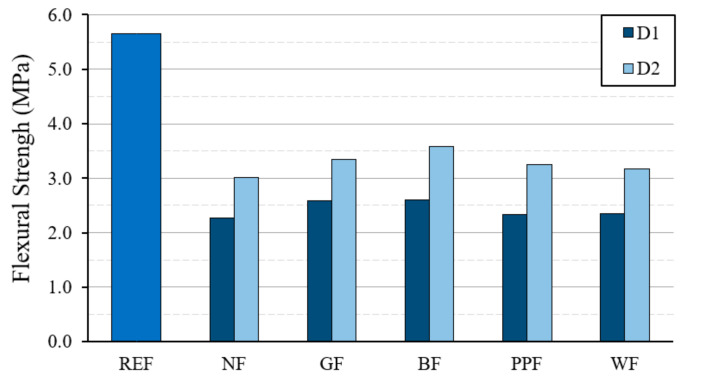
Results of the flexural strength test on standard 4 × 4 × 16 cm^3^ specimens.

**Figure 7:**
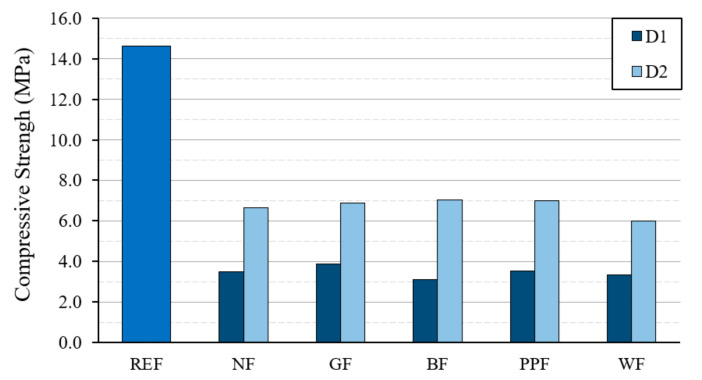
Results of the compressive strength test on standard 4 × 4 × 16 cm^3^ specimens.

Figure 6 and Figure 7 show the results obtained for the values of the mechanical resistance to flexural traction and compression of the different dosages used. These results correspond to the tests carried out on standard RILEM 4 × 4 × 16 cm^3^ specimens.

As can be seen in Figure 6, the new lightened plaster material presents lower flexural strength values than the reference test piece, with the D2 type dosage obtaining better performance compared to the D1 type dosage. It can be seen how the use of reinforcing fibers makes it possible to slightly improve the flexural strength and delay the effect of superficial cracks in accordance with previous studies [53,54]. The batches with the incorporation of basalt fiber were the ones that presented higher resistance values compared to the other reinforcing fibers used. However, in all cases, strengths greater than 2 MPa were always obtained. After this test, although all specimens with fiber improved their strength, synthetic fibers were better than natural wood fiber.

Regarding the compressive strengths of the designed plaster material shown in Figure 7, it can be seen that the mixes made with the type D2 dosage also presented higher values than the type D1 mixes. The incorporation of fibers did not cause a significant improvement in the resistance to compression, although it can be observed how the kneaded mixture with the addition of synthetic fibers obtained values slightly higher than the dosages with the incorporation of wood fiber. In any case, all the dosages used exceeded 3 MPa of compressive strength.

**Figure 8:**
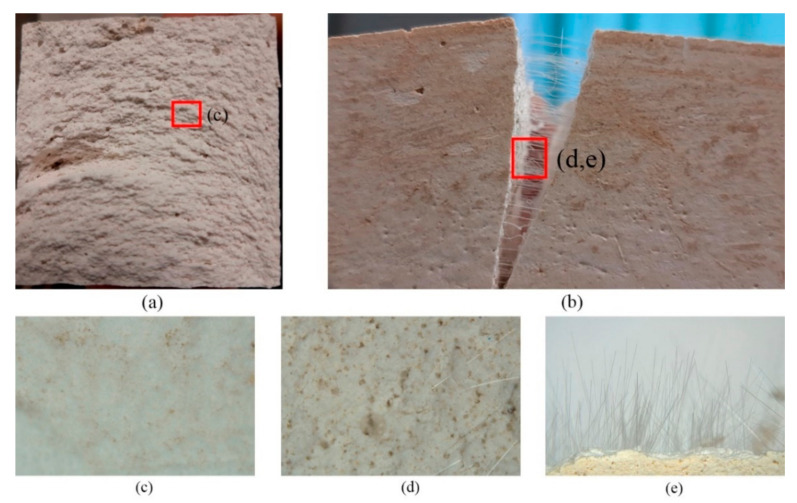
Specimen type D1 (**a**) and D1-PPF (**b**) and different images taken by light microscope (**c**–**e**).

Figure 8 shows some images obtained by scanning electron microscopy. These are presented to help understand the internal structure of the material developed in this investigation.

Figure 8a shows the section of a lightened specimen obtained after carrying out the flexural test. It is clear how the release of CO_2_, generated from sodium bicarbonate in an acid medium provided by the boric acid solution, favors the formation of a banded structure which grows convexly and perpendicular to the surface. This anisotropic structure generates preferential directions of failure in flexural and compression tests. Figure 8c shows a microscopic enlargement of the surface of the material, where its soapy texture can be appreciated. Figure 8b,d,e show the flexural break of a specimen lightened and reinforced with polypropylene fibers. It can be observed how the material remains together once the break occurs as a result of the internal cohesion caused by the fibers, and also the good adhesion between the lightened plaster matrix and the reinforcing fibers.

**Figure 9:**
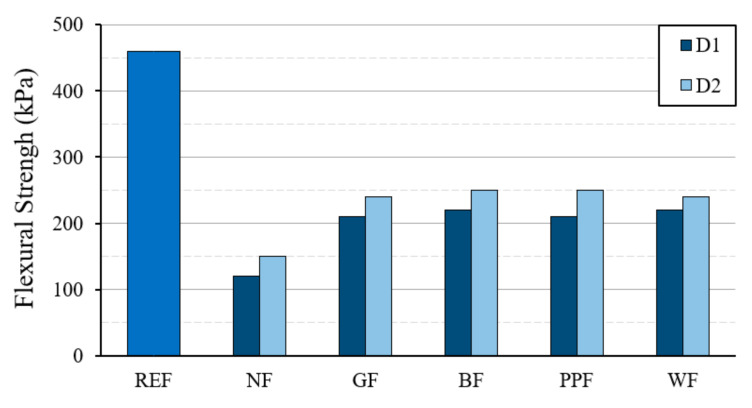
Results of flexural strength in 50 × 30 × 2 cm^3^ samples.

On the other hand, Figure 9 shows the results derived from the pure bending test on plates with dimensions of 50 × 30 × 2 cm^3^. These tests indicate the technical feasibility of the plaster material developed to be used in the preparation of precasts, which, together with its lower density compared to traditional plaster panels, improves labor yields. In this way, the typology of prefabricated works elaborated in this study reduces costs and execution times [55].

As can be seen in Figure 9, the incorporation of reinforcing fibers in the plaster matrix has a positive effect on the mechanical behavior of the lightened plaster material, which is in agreement with the results obtained by other researchers [56]. It can be seen that in all the dosages made with the incorporation of fibers, the flexural strength exceeded 200 kPa. In addition, in the specimens made with these dimensions, the differences between strengths of the D1 and D2 type dosages were less pronounced than in the previous flexural and compression tests, obtaining slightly higher results for the D2 type dosage. The reference dosage reached values of flexural strength of 460 kPa, a value three times higher than that reached in the lightened plaster material without fiber reinforcement. Having obtained these results, it is possible to develop plasterboards and ceilings for their use in buildings.

**Figure 10:**
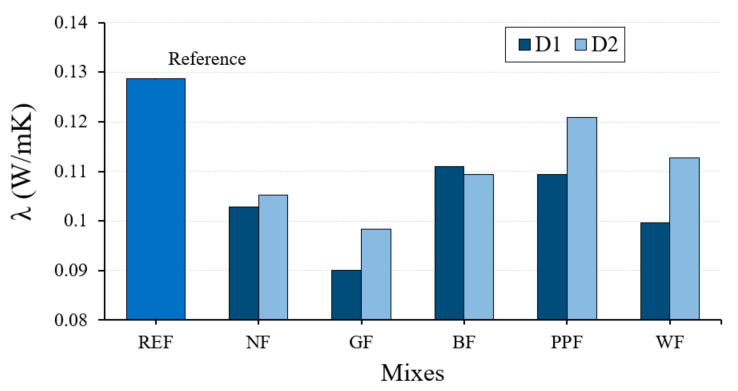
Results of the thermal conductivity obtained for the different dosages studied.

Another of the most important qualities of plaster materials is related to comfort and habitability [55]. In Figure 10, the results obtained for the thermal conductivity values in the different dosages used are shown.

Figure 10 shows how the thermal conductivity of all the dosages made with the lightened plaster material were lower than those obtained by the reference plaster dosage. Type D1 dosages presented lower thermal conductivity values than type D2 dosages. The best results were obtained in the D1-GF type dosages, whose thermal conductivity value is up to 30% lower than that obtained for the reference plaster material. However, it should be noted that, except for this case, lightened specimens without fibers have lower thermal conductivity values than specimens containing fibers.

Finally, the mechanical and thermal properties of the D1 and D2 series can be explained based on the same concepts of the porous structure already presented. Thus, the higher porosity already mentioned in the samples with PVAc, sodium bicarbonate and boric acid compared to the reference would account for the lower mechanical properties, lower surface hardness and lower thermal conductivity, with the decrease in these properties being more pronounced in the D1 series that incorporates a higher quantity of all of these compounds in the formulation.

### 3.3. Statistical Analysis

To carry out the statistical analysis, the suitability of the sample for an analysis of variance (ANOVA) must be verified. The assumptions to be tested to apply an ANOVA are: (1) the K samples are independent; (2) the populations all have the same variance (homoscedasticity); and (3) the populations present a normal probability distribution [56]. The results obtained are shown in Table 7.

As can be seen in Table 7, it has been verified whether the properties to be included in the analysis maintain a normal probability distribution using the Kolmogorov–Smirnov test. It is clear that, with the exception of thermal conductivity, the test statistic presents statistical significance (*p* < 0.001), so the null hypothesis is rejected, and it is stated that adherence, density, surface hardness, resistance to compression and flexural strength do not meet the assumptions for performing the ANOVA analysis. Next, the homoscedasticity of the thermal conductivity variable has been verified using the Levene statistic, which, as can be seen in Table 8, is statistically significant (*p* < 0.001), so the assumption of equality of variances of the variable studied is rejected.

Following this verification, it was concluded that to be able to compare the means of the different properties studied in each of the dosages used, it is necessary to use non-parametric tests [57]. In this case, the Kruskal–Wallis test has been used for independent samples.

Table 8 shows the confidence intervals for each dosage and property studied. The dosages with the best results are indicated with the symbol (↑). This was verified with the mean values for which there are statistically significant differences, indicated with (*). In the adherence property, D1-NF presents the highest mean value (0.58 MPa), and the difference of this value with the mean obtained by D1-BF (0.15 MPa) is statistically significant for a confidence level greater than 99% according to the results of the Kruskal–Wallis test. In the case of density, the best value is obtained by D1-NF (842.98 kg/m^3^), although N1-PPF manages to reduce the value further (840.50 kg/m^3^), a difference that is not statistically significant. Regarding hardness, the best result is obtained for D2-NF (74.98 Ud. Shore C), although D2-BF and D2-WF exceeded this value without the differences between the means being statistically significant. The same occurs with the compressive strength, as the best result was obtained for D2-NF (6.60 MPa), surpassed by D2-BF (7.07 MPa) and D2-PPF (7.01 MPa), but the differences observed in Table 8 are not statistically significant. Regarding the flexural strength, the best result is presented by D2-NF (3.02 MPa), being exceeded by all the dosages with the incorporation of fibers without these differences being statistically significant. Finally, regarding thermal conductivity, the best result is presented by D1-NF (0.10 W/mK), improved only by D1-GF (0.09 W/mK), although this difference is not statistically significant.

## 4. Conclusions

In this work, a new lightened plaster material has been presented that reduces density by between 12% and 23% compared to traditional plaster materials. This decrease in the density of the material, together with its longer starting time for setting, make it an ideal alternative to produce precast plaster in buildings. On the other hand, the material designed in this research has obtained better thermal properties compared to traditional plaster material, where values for the coefficient of thermal conductivity λ between 0.09 and 0.12 W/mK have been obtained. These results reflect the good thermal behavior of the lightened plaster and its suitability for achieving greater thermal comfort through its use in interior partitions and false ceilings.

With respect to the mechanical properties of the material, it is possible to appreciate a decrease in resistance compared to the reference plasters, without this affecting its application as a material for the execution of precast and interior coatings. Regarding adhesion, higher values have been obtained in mixes with the incorporation of reinforcing fibers, reaching values of up to 0.58 MPa in type D1 dosages that are very similar to those obtained for traditional plaster. Regarding the surface hardness, although this decreased with the incorporation of the polymeric compound, the lowest value was 65 Ud. Shore C.

These properties have been explained based on the formation of a porous structure in the material due to two overlapping contributions. On one hand, there is the generation of CO_2_ bubbles, due to the reaction of sodium bicarbonate and boric acid, that generate voids in the specimen. On the other hand, there is the formation of a three-dimensional network of cross-linked polymeric chains caused by the reaction of the borate ions with the polyvinyl polymer, which could also contribute to the generation of porosity. The formation of this network, in which part of the mixing water would be trapped, would delay the start of the setting reaction (dihydrate formation). The network, once formed and the drying process of the specimens has ended, causes the formation of a dihydrate crystal skeleton, with fewer connections between them than in the reference specimen, and therefore greater porosity. These characteristics would yield a lower apparent density, lower thermal conductivity, lower surface hardness and lower mechanical properties for the specimens with added polymer and boric acid, and to a greater degree in the specimens with a higher content of these components.

The flexural strengths reflect that it is possible to use this material in the elaboration of precasts such as false ceilings. In all cases tested with standard RILEM specimens, values greater than 2 MPa were obtained. A positive effect of the addition of fibers in the plaster matrix to improve this property has been appreciated, reaching values greater than 3.5 MPa for D2-BF type dosages. In addition, when testing boards measuring 50 × 30 × 2 cm^3^, although a decrease in mechanical resistance is observed, all the lightened fiber-reinforced plasters obtained values higher than 200 kPa. Synthetic fiber has been shown to be a better reinforcing material than natural wood fiber in flexural strength. Basalt fiber was the best reinforcing fiber. With respect to compressive strength, the incorporation of reinforcing fibers does not present a special increase in resistance, and in all the cases tested, values greater than 3 MPa were reached for the D1 type dosages and 6 MPa for the type D2 dosages.

As a future line of research, the authors want to highlight the need to carry out tests that allow us to evaluate the behavior of the lightened plaster material. In this way, its suitability to be used on interior walls would be evaluated. The aim is to check the suitability to hang furniture elements.

Finally, it is convenient to highlight that the research presented in this work is the consequence of patent ES 2 722 598 B2 obtained by the authors, entitled: “*Material de escayola aligerada con polímeros para uso en placas y panels prefabricados*” [58].

## Figures and Tables

**Table 1:** Relationship between tests and samples used.

Sample *	Tests
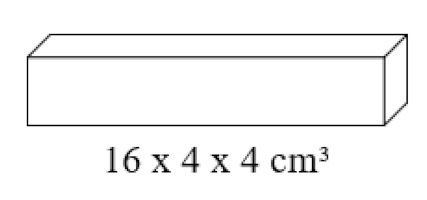	Surface hardness using a Shore C durometer, bulk density according to the recommendations of the UNE 102042: 2014 standard using the weight of the specimen seven days after it was done and 4 × 4 × 16 cm^3^ volume [36]. Resistance to flexural traction and compression with the help of an AUTOTEST 200-10SW, (Madrid, Spain) model hydraulic press according to the recommendations of the UNE-EN-13279-2: 2014 standard [37].
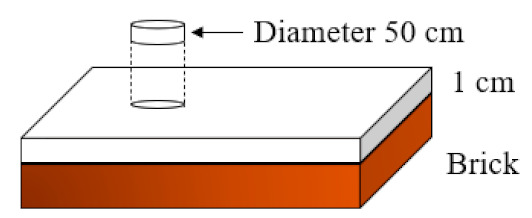	Test for the measurement of adherence of lightened plaster material on a previously moistened brick masonry base in accordance with the UNE-EN-13279-2: 2014 standard [37].
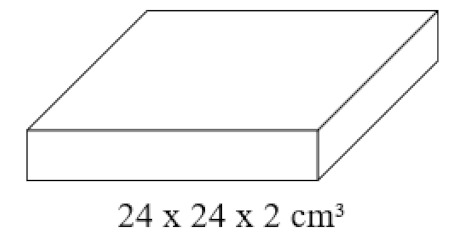	Determination of the coefficient of thermal conductivity of the material with the help of a thermal box. The specimens were tested for a period of 12 h until the heat flux across the surface remained steady.
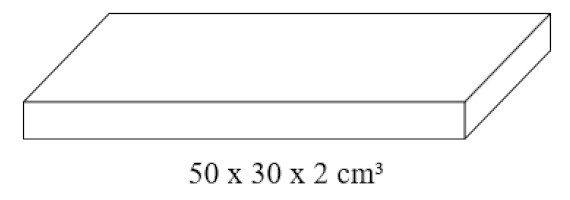	Pure bending test to determine the suitability of the material for use in precasts. For this, Proeti, S.A., (Madrid, Spain) ending test equipment was used and the recommendations of the standard UNE-EN 12859: 2012 [38] were followed.

* Three specimens of each type have been tested for each dosage and for each of the tests carried out.

**Table 2:** Iberyola E-35 properties [15].

Normative	Thermal Conductivity	Water Vapor Diffusivity (μ)	Purity Index (%)	Flexural Strength
EN-12379	0.30 W/mK	6	>90	>3 MPa
**Color**	**Fire Reaction**	**Granulometry**	**Water/Binder Ratio**	**pH**
White	A1 *	0–0.2 mm	0.7–0.8	>6

* Fire resistance according to the Technical Building Code, document CTE-DB: SI [37].

**Table 3:** Mechanical properties of used fibers.

Fiber	Density (kg/m^3^)	Modulus of Elasticity (GPa)	Traction Strength (GPa)	Elongation to Break (%)	Length (mm)
Polypropylene	910	6	0.4	80–140	12
Basalt	2750	91	4	1.8	
Glass	2680	72	1.7	4.3	
Wood	589	12	7 *	30–50	

* Measured in the parallel direction to the fiber.

**Table 4:** Dosages used. PVAc—polyvinyl acetate

Type	Components				
	PVAc (g)	Boric Acid Solution (g)	NaHCO_3_ (g)	Water (g)	Plaster (g)
REF	-	-	-	660	1000
D1	100	50	7.5	660	1000
D2	75	37.5	5.625	660	1000
Fibers	NF (g)	GF (g)	BF (g)	PPF (g)	WF (g)
Amount	0	2.5	2.5	2.5	7.0

**Table 5:** Setting start time of the dosages used.

Sample	REF	D1	D2
Initial setting time (min)	13	22	20

**Table 6:** Adhesion, surface hardness and bulk density of the different specimens. NF—no fibers, GF—glass fibers, BF—basalt, PPF—polypropylene fibers, WF—wood fibers.

Sample	Adhesion (MPa)	Surface Hardness (U. Shore C)	Bulk Density (kg/m^3^)	Bulk Density Variation with Respect to the Reference Batch (%)
REF	0.62	87	1102.6	-
D1–NF	0.58	65	843.0	↓ 23.54
D2-NF	0.29	75	937.7	↓ 14.96
D1-GF	0.22	69	849.0	↓ 23.00
D2-GF	0.18	75	960.8	↓ 12.86
D1-BF	0.17	67	850.6	↓ 22.85
D2-BF	0.14	77	960.4	↓ 12.90
D1-PPF	0.19	67	840.5	↓ 23.77
D2-PPF	0.15	75	959.1	↓ 13.01
D1-WF	0.20	66	844.9	↓ 23.37
D2-WF	0.18	77	966.1	↓ 12.40

**Table 7:** Suitability of assumptions for ANOVA analysis.

Property	Kolmogorov–Smirnov	Homocedasticity
	Statistic	Levene Statistic
Adhesion	0.296 ***	-
Bulk density	0.245 ***	-
Surface hardness	0.149 **	-
Compressive strength	0.245 ***	-
Flexural strength	0.196 ***	-
Thermal conductivity	0.099	5.018 ***

Note. ** *p* < 0.01, *** *p* < 0.001.

**Table 8:** Confidence intervals and standard deviation (σ) for the mean for each dosage and property.

**Sample**	**Adhesion (MPa)**		**Bulk Density (kg/m^3^)**		**Hardness (Ud. Shore C)**	
	**95% CI**	**σ**	**95% CI**	**σ**	**95% CI**	**σ**
REF	(0.61, 0.63)	0.01	(1102.20, 1103.01)	0.38	(86.49, 87.49)	0.61
D1-NF	(0.56, 0.60) ^↑^	0.02	(841.46, 844.51) ^↑^	1.45	(62.02, 67.91)	2.80
D2-NF	(0.26, 0.32)	0.03	(935.93, 939.63)	1.76	(74.27, 75.69) ^↑^	0.68
D1–GF	(0.21, 0.23)	0.01	(846.37, 851.82)	2.60	(67.61, 69.36)	0.84
D2–GF	(0.16, 0.20)	0.02	(956.39, 965.22)	4.21	(74.14, 76.03)	0.90
D1–BF	(0.13, 0.17) *	0.02	(847.67, 853.68)	2.87	(66.11, 68.26)	1.02
D2–BF	(0.10, 0.14)	0.02	(960.01, 960.81)	0.38	(75.78, 78.09)	1.10
D1–PPF	(0.16, 0.22)	0.03	(834.50, 846.50)	5.72	(66.18, 68.12)	0.93
D2–PPF	(0.12, 0.18)	0.03	(956.36, 961.86)	2.62	(74.32, 76.12)	0.86
D1–WF	(0.19, 0.21)	0.01	(843.10, 846.73)	1.73	(65.81, 68.49)	1.28
D2–WF	(0.16, 0.20)	0.02	(964.03, 968.21)	1.99	(76.31, 77.29)	0.47
**Sample**	**Flexural Strength (MPa)**		**Compressive Strength (MPa)**		**λ (W/mK)**	
	**95% CI**	**σ**	**95% CI**	**σ**	**95% CI**	**σ**
REF	(5.34, 5.96)	0.30	(14.13, 15.03)	0.43	(0.13, 0.13)	0.00
D1-NF	(2.19, 2.35)	0.08	(3.31, 3.65)	0.16	(0.10, 0.10) ^↑^	0.00
D2-NF	(2.88, 3.16) ^↑^	0.13	(6.35, 6.84) ^↑^	0.23	(0.10, 0.11)	0.00
D1–GF	(2.35, 2.81)	0.22	(3.11, 4.56)	0.69	(0.08, 0.10)	0.01
D2–GF	(3.21, 3.47)	0.12	(6.26, 7.40)	0.54	(0.09, 0.10)	0.01
D1–BF	(2.44, 2.76)	0.15	(2.43, 4.01)	0.76	(0.10, 0.12)	0.01
D2–BF	(3.51, 3.64)	0.06	(6.87, 7.27)	0.19	(0.10, 0.12)	0.01
D1–PPF	(2.14, 2.53)	0.19	(3.37, 3.73)	0.17	(0.10, 0.12)	0.01
D2–PPF	(3.17, 3.34)	0.08	(6.90, 7.11)	0.10	(0.12, 0.12)	0.00
D1–WF	(2.26, 2.42)	0.08	(3.20, 3.46)	0.13	(0.09, 0.10)	0.00
D2–WF	(3.06, 3.29)	0.11	(5.68, 6.24)	0.27	(0.11, 0.11)	0.00

Note: ^↑^ Best value for the property studied between D1-NF and D2-NF, * statistically different for 99% confidence level.

## Data Availability

Not applicable.

## References

[1] Villanueva L., García-Santos A. (2001). Manual del Yeso.

[2] Ferrández D., Morón C., Saiz P., Atanes-Sánchez E., Yedra E. (2020). Low-Cost Sensors for Determining the Variation in Interior Moisture Content in Gypsum Composite Materials. Materials.

[3] Krejsova J., Dolezelova M., Pernicova R., Svora P., Vimmrova A. (2018). The influence of different aggregates on the behavior and properties of gypsum mortars. Cem. Concr. Compos..

[4] Oliver A., Neila F.J., García A. (2010). Caracterización térmica de placas de yeso con material de cambio de fase incorporado. Inf. Construcción.

[5] Tejela J., de Artega Garrifo I. (2011). Prefabricado de Placas de Yeso Laminado.

[6] Pedreño-Rojas M.A., Morales-Conde M.J., Pérez-Galvéz F., Rodríguez-Linan C. (2017). Eco-efficient acoustic and thermal conditioning using false ceiling plates made from plaster and wood waste. J. Clean. Prod..

[7] Fenoglio E., Fantucci S., Serra V., Carbonaro C., Pollo R. (2018). Hygrothermal and environmental performance of a perlite-based insulating plaster for the energy retrofit of buildings. Energy Build..

[8] Gencel O., del Coz J.J., Sutcu M., Kokal F., Álvarez F.P., Martínez-Barrera G. (2016). A novel lightweight gypsum composite with diatomite and polypropylene fibers. Constr. Build. Mater..

[9] García A. (2018). Material de Construcción de Yeso o Escayola Aligerado y Su Uso en Placas y Paneles.

[10] Catalán F.J. (2015). Placa de Yeso Laminado Aligerada con Corcho Granulado.

[11] Bicer A., Kar F. (2017). Thermal and mechanical properties of gypsum plaster mixed with expanded polystyrene and tragacanth. Therm. Sci. Eng. Prog..

[12] Evans T.J., Majumdar A.J., Ryder F.J. (1981). A semi-dry method for the production of lightweight glass-fibre-reinforced gypsum. Int. J. Cem. Compos. Lightweight Concr..

[13] Dima C., Badanoiu A., Cirstea S., Nicoara A.I., Stoleriu S. (2020). Lightweight Gypsum Materials with Potential Use for Thermal Insulations. Materials.

[14] Gutiérrez-González S., Gadea J., Rodríguez A., Blanco-Varela M.T., Calderón V. (2012). Compatibility between gypsum and polyamide powder waste to produce lightweight plaster with enhanced thermal properties. Constr. Build. Mater..

[15] Vidales A. (2019). Caracterización Fisicoquímica y Aplicaciones de Yeso con Adición de Residuo Plástico de Cables Mediante Criterios de Economía Circular. Ph.D. Thesis.

[16] Gutiérrez-González S., Gadea J., Rodríguez A., Junco C., Calderón V. (2012). Lightweight plaster materials with enhanced thermal properties made with polyurethane foam wastes. Constr. Build. Mater..

[17] Di Bella G., Fiore V., Galtieri G., Borsellino C., Valenza A. (2014). Effects of natural fibres reinforcement in lime plasters (kenaf and sisal vs. Polypropylene). Constr. Build. Mater..

[18] Ashour T., Wieland H., Georg H., Bockisch F.-J., Wu W. (2020). The influence of natural reinforcement fibres on insulation values of earth plaster for straw bale buildings. Mater. Des..

[19] Vega D.F. (2016). Estudio de la Transmisión de Vibraciones por Impacto en Losas de Hormigón y Mortero. Ph.D. Thesis.

[20] Álvarez M., Feroldi S. Characterization of High Isolation Gypsum. Proceedings of the IV International Conference on Technological Innovation in Building, CITE 2019.

[21] Dai D., Fan M. (2015). Preparation of bio-composite from wood sawdust and gypsum. Ind. Crop. Prod..

[22] Bustos Garcia A. (2018). Morteros con Propiedades Mejoradas de Ductilidad por Adición de Fibras de Vidrio, Carbono y Basalto. Ph.D. Thesis.

[23] Iucolano F., Boccarusso L., Langella A. (2019). Hemp as eco-friendly substitute of glass fibres for gypsum reinforcement: Impact and flexural behaviour. Compos. Part B Eng..

[24] Quintana A., Alba J., del Rey R., Guillén I. (2018). Comparative Life Cycle Assessment of gypsum plasterboard and a new kind of bio-based epoxy composite containing different natural fibers. J. Clean. Prod..

[25] Binici H., Aksogan O. (2017). Insulation material production from onion skin and peanut shell fibres, fly ash, pumice, perlite, barite, cement and gypsum. Mater. Today Commun..

[26] Huang X. (2009). Fabrication and Properties of Carbon Fibers. Materials.

[27] Suárez F., Felipe-Sesé L., Diaz F.A., Gálvez J.C., Alberti M.G. (2020). On the fracture behaviour of fibre-reinforced gypsum using micro and macro polymer fibres. Constr. Build. Mater..

[28] Zhu C., Zhang J., Peng J., Cao W., Liu J. (2018). Physical and mechanical properties of gypsum-based composites reinforced with PVA and PP fibers. Constr. Build. Mater..

[29] Medina N.F., Barbero-Barrera M.M. (2017). Mechanical and physical enhancement of gypsum composites through a synergic work of polypropylene fiber and recycled isostatic graphite filler. Constr. Build. Mater..

[30] Bustos A., Cobo A., Yunta F., Moreno E. (2018). Influence of fibers addittion on properties of hydraulic lime based mortars. DYNA.

[31] Del Rio Merino M., Olivares F.F. (2004). Escayola aligerada: Propuestas alternativas a la adición de sólidos celulares. Mater. Construcción.

[32] Del Rio Merino M., Astorqui J.S.C., Olivares F.F. (2005). New prefabricated elements of lightened plaster used for partitions and extrados. Constr. Build. Mater..

[33] Sáez P.V., Del Rio Merino M., Sorrentino M., Amores C.P., Astorqui J.S.C., Arrebola C.V. (2020). Mechanical Characterization of Gypsum Composites Containing Inert and Insulation Materials from Construction and Demolition Waste and Further Application as A Gypsum Block. Materials.

[34] Sair S., Mandili B., Taqi M., El Bouari A. (2019). Development of a new eco-friendly composite material based on gypsum reinforced with a mixture of cork fibre and cardboard waste for building thermal insulation. Compos. Commun..

[35] Strydom C.A., Potgieter J.H. (1999). Dehydration behaviour of a natural gypsum and a phosphogypsum during milling. Thermochim. Acta.

[36] Spanish Association for Standardization (2014). UNE-EN-102042:2014. Gypsum Plasters. Other Test Methods.

[37] Spanish Association for Standardization (2014). UNE-EN-13279-2:2014. Gypsum Binders and Gypsum Plasters—Part 2: Test Methods.

[38] Spanish Association for Standardization (2012). UNE-EN 12859:2012. Gypsum Blocks—Definitions, Requirements and Test Methods.

[39] Fleck W.E.P., Jones M.H., Kuntze R.A., McAdie H.G. (1960). The Differential Thermal Analysis of Natural and Synthetic Hydrates of Calcium Sulphate. Can. J. Chem..

[40] Thermal Analysis Application (2010). Determination of Calcium Sulfate Dihydrate and Hemihydrate in Cement.

[41] González A.D.S.A. (2017). Caracterización de Compuestos Eco-Eficientes de Yeso Aligerado con Residuo de Poliestireno Extruido (XPS). Ph.D. Thesis.

[42] Santos A.G. (1988). Comportamiento mecánico de yeso reforzado con polímeros sintéticos. Inf. Constr..

[43] Angelova L.V., Terech P., Natali I., Dei L., Carretti E., Weiss R.G. (2011). Cosolvent Gel-like Materials from Partially HydrolyzedPoly(vinyl acetate)s and Borax. Langmuir.

[44] Militiký J., Kovacic V., Rubnerova J. (2002). Influence of thermal treatment on tensile failure of basalt fibers. Eng. Fract. Mech..

[45] Wu Y.-F. (2009). The structural behavior and design methodology for a new building system consisting of glass fiber reinforced gypsum panels. Constr. Build..

[46] Fernandez C.M., Vega D.F., Martinez P.S., Martinez F.F. (2019). Behaviour of masonry mortars fabricated with recycled aggregate towards moisture. DYNA.

[47] Canal de Isabel II (2012). Informe Anual Sobre la Calidad del Agua en Madrid.

[48] Sanz Arauz D. (2009). Análisis del Yeso Empleado en Revestimientos Exteriores Mediante Técnicas Geológicas. Ph.D. Thesis.

[49] Barrera J.E., Rodríguez J.A., Perilla J.E., Algecira N.A. (2007). Estudio de la degradación térmica del poli(alcohol vinílico) mediante termogravimetría y termogravimetría diferencial. Rev. Ing. E Investig..

[50] Vega D.F., Alvarez E.Y., Fernandez C.M., Barrios A.M. (2020). Alternative test for the determination of the setting time. Capacitive and resistive methods. DYNA.

[51] Samide A., Tutunaru B., Merisanu C., Cioatera N. (2020). Thermal analysis: An effective characterization method of polyvinyl acetate films applied in corrosion inhibition field. J. Therm. Anal. Calorim..

[52] Dharmasastha K., Samuel D.L., Nagendra S.S., Maiya M. (2020). Experimental investigation of thermally activated glass fibre reinforced gypsum roof. Energy Build..

[53] Romero-Gómez M.I., Pedreño-Rojas M.A., Pérez-Galvez F., Rubio-de-Hita P. (2021). Characterization of gypsum composites with polypropylene fibers from non-degradable wet wipes. J. Build. Eng..

[54] Zhai Y., Xu G., Huang G.Q. (2019). Buffer space hedging enabled production time variation coordination in prefabricated construction. Comput. Ind. Eng..

[55] Srinivasaraonaik B., Singh L.P., Shina S., Tyagi I., Rawat A. (2020). Studies on the mechanical properties and thermal behavior of microencapsulated eutectic mixture in gypsum composite board for thermal regulation in the buildings. J. Build. Eng..

[56] Peña D. (2010). Regresión y Diseño de Experimentos.

[57] Theodorsson-Norheim E. (1986). Kruskal-Wallis test: BASIC computer program to perform nonparametric one-way analysis of variance and multiple comparisons on ranks of several independent samples. Comput. Methods Programs Biomed..

[58] Ferrández D., Morón C., Álvarez M., Saiz P. (2019). Material de Escayola Aligerada con Polímeros para Uso en Placas y Paneles Prefabricados.

